# A Non-Contact Measurement System for the Range of Motion of the Hand

**DOI:** 10.3390/s150818315

**Published:** 2015-07-28

**Authors:** Trieu Pham, Pubudu N. Pathirana, Hieu Trinh, Pearse Fay

**Affiliations:** 1 School of Engineering, Faculty of Science, Engineering & Built Environment, Deakin University, 75 Pigdons Road, Waurn Ponds, Victoria 3216, Australia; E-Mails: pubudu.pathirana@deakin.edu.au (P.N.P.); hieu.trinh@deakin.edu.au (H.T.); 2 School of Health and Social Development Occupational Therapy, Deakin University, 1 Gheringhap Street, Geelong, Victoria 3220, Australia; E-Mail: pearse.fay@deakin.edu.au

**Keywords:** non-contact measurement, range of motion, hand, total active movement

## Abstract

An accurate and standardised tool to measure the active range of motion (ROM) of the hand is essential to any progressive assessment scenario in hand therapy practice. Goniometers are widely used in clinical settings for measuring the ROM of the hand. However, such measurements have limitations with regard to inter-rater and intra-rater reliability and involve direct physical contact with the hand, possibly increasing the risk of transmitting infections. The system proposed in this paper is the first non-contact measurement system utilising Intel Perceptual Technology and a Senz3D Camera for measuring phalangeal joint angles. To enhance the accuracy of the system, we developed a new approach to achieve the total active movement without measuring three joint angles individually. An equation between the actual spacial position and measurement value of the proximal inter-phalangeal joint was established through the measurement values of the total active movement, so that its actual position can be inferred. Verified by computer simulations, experimental results demonstrated a significant improvement in the calculation of the total active movement and successfully recovered the actual position of the proximal inter-phalangeal joint angles. A trial that was conducted to examine the clinical applicability of the system involving 40 healthy subjects confirmed the practicability and consistency in the proposed system. The time efficiency conveyed a stronger argument for this system to replace the current practice of using goniometers.

## 1. Introduction

**Figure 1:**
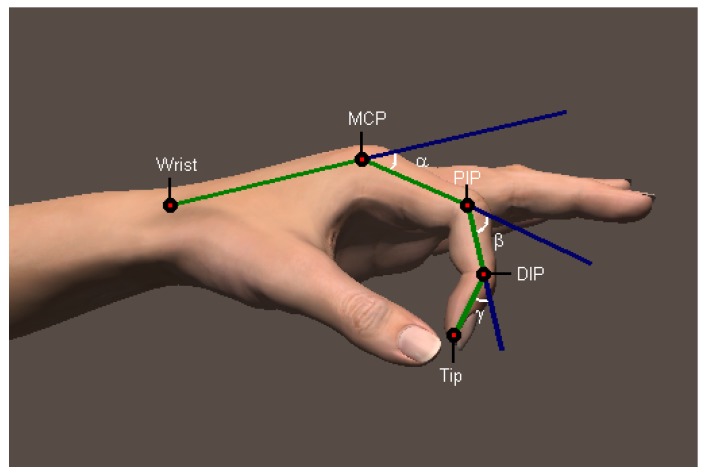
Position of the phalangeal joints.

Our hands play a pivotal role in performing daily activities and interacting with the surrounding world. Understanding the way that our hands move provides insight into how daily activities are performed, an integral part in rehabilitation following hand injuries. Measuring the phalangeal range of motion (ROM) is an essential part in clinical practices. Medical professionals often use universal goniometers, inclinometers or electro-goniometers to measure the declination angles of finger joints to assess the joint movement range [1,2,3]. These joints involve four main bones for each finger: metacarpal, proximal phalanx, middle phalanx and distal phalanx. The joint between the metacarpal and proximal phalanx is named the metacarpophalangeal joint (MCP). The joint between the proximal phalanx and the middle phalanx is called the proximal interphalangeal joint (PIP), while the joint between the middle phalanx and the distal phalanx is called the distal interphalangeal joint (DIP). Note that this description is not true for the thumb. The thumb does not possess a middle phalanx; hence, it has an MCP and an interphalangeal joint (IP). The position of each joint and the angles of interest of the finger joints are depicted in Figure 1.

Historically, subjective visual examinations have been used to examine the ROM, and subsequently, the declination joint angles were measured with universal goniometers to evaluate ROM [4]. With the development of technology, new goniometer models were gradually introduced and improved to assist clinicians [5,6,7,8,9,10,11,12,13,14]. In measuring rotary motions of the forearm and shoulder, Laupattarakasem *et al.* [5] introduced an axial rotation gravity goniometer to improve reliability. In another research work, one of the first two-element optic fibre goniometers was built using graded-index microlens receivers [6]. The fibre goniometer was improved in a later study by Donno *et al.* [10]. When personal computers became popular and were capable of effortlessly communicating with a variety of hardware, Barreiro *et al.* built a computer-based goniometer, which can directly record declination angle on a personal computer [8]. Researchers also wanted to reduce the production cost of goniometers, such as in Coburn *et al.*’s study [9], wherein they used remote sensors to build a goniometer. In more recent research, the development of MoCap (Motion Capture) systems provided a convenient and accurate approach to evaluate ROM, such as the use of a Vicon system in Windolf *et al.*’s study [13] and a Kinect-based system in Pfister *et al.*’s study [14]. More interestingly, smart phones with integrated accelerometer sensors have also been considered [11].

One of the challenges in the current practice is that the assessment tools, including universal goniometer, electro-goniometer, optical fibre goniometer, Vicon and accelerometer integrated smart phones, require physical contact with the finger to achieve the best accuracy. However, injuries, such as burns, wounds, lacerations or even dermatological conditions, can cause difficulties with the assessment tool, due to bandages, the risk of infection or discomfort. When clinicians align goniometers along phalangeal bones, they need to maintain a small gap with the skin or to place the tool on top of the bandage. Both ways are inconvenient and tend to be subjective and error prone. Another significant challenge is intra- and inter-rater reliability [15]. Studies into the reliability of universal goniometer report a variance of 7${}^{\circ }$–9${}^{\circ }$ between therapists [15,16] when measuring joint angles, leading to a 27${}^{\circ }$ difference over the three joints of a finger. Research has been conducted on the reliability of universal goniometers and proposed devices [11,12,17,18,19,20,21,22], as reliability is an important aspect in clinical practice.

**Figure 2:**
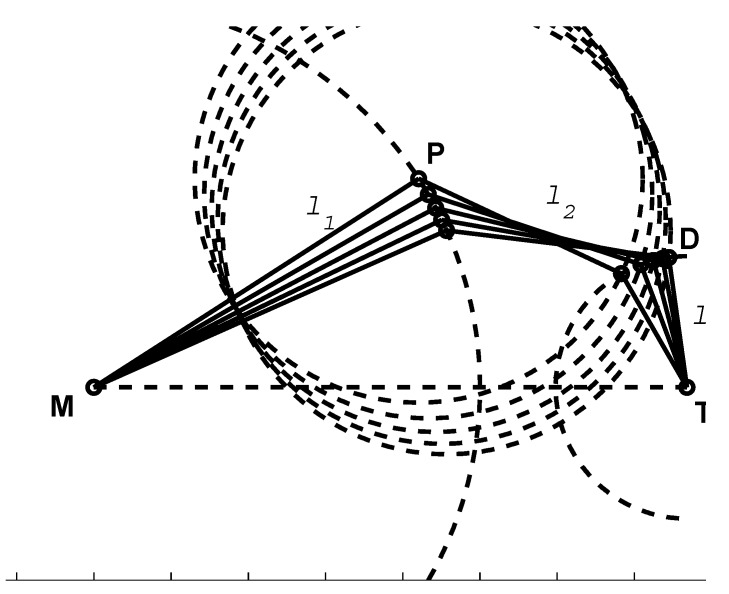
For a fixed base position of the metacarpal phalangeal joint M, there are infinitely many solutions of ${\text{θ}}_{MCP}$, ${\text{θ}}_{PIP}$ and ${\text{θ}}_{DIP}$, or in other words, **P** and **D**, to place the fingertip **T** into a particular position, in addition to the knowledge of the lengths of the phalangeal bones ($l_{1}$, $l_{2}$ and $l_{3}$).

Adapting optical measurement systems or computer vision-based approaches provides a non-contact form of measurement that can address the current challenges. In recent years, captures of hand movements have attracted attention, particularly with the development of a number of pervasive devices, such as the Microsoft Kinect Sensor and the Leap Motion Controller, as they offer better solutions in measuring both body and finger movements [23]. More recent research in this area used a Microsoft Kinect${}^{©}$ to build a 3D skeletal hand tracking system [24,25]. Metcalf *et al.* [26] have recently proposed a Kinect-based system to capture motion and to measure hand kinematics. However, Kinect${}^{©}$ is primarily aimed at full body movements and has limitations concerning the accuracy required for finer finger movements. Although the study claims an accuracy of less than $15^{\circ }$, the system is not reliable to use in medical and rehabilitation applications due to a lack of an appropriate level of confidence in the measurements. While developing the system, Metcalf introduced a hypothesis that for a fixed base position of the metacarpophalangeal (MCP) joint, there will be only one combination of ${\text{θ}}_{MCP}$, ${\text{θ}}_{PIP}$ and ${\text{θ}}_{DIP}$ to place the fingertip into a particular position. While this hypothesis was experimentally verified in their paper, the trial was limited to only healthy personnel. There is no guarantee that all of the joints and muscles in an injured hand will follow regular movements and constraints. Moreover, it is clear that the hypothesis failed on its mathematical standpoint (Figure 2). The Leap Motion Controller is a promising device for hand gesture recognition and fingertip tracking, but lacks inter-phalangeal joint detection capability necessary for the underlying medical application. In its hand tracking model, the proximal inter-phalangeal joint and the distal inter-phalangeal joint are estimated based on the constraint ${\text{θ}}_{DIP}=\frac{2}{3}{\text{θ}}_{MCP}$, which is defined only for healthy hands. As reported by ElKoura *et al.* [27], this constraint is a rough approximation for the intricate control of the hand. In his experiments with 20,000 samples, the difference of the value ${\text{θ}}_{DIP}-\frac{2}{3}{\text{θ}}_{MCP}$ ranges from $-36^{\circ }$–$42^{\circ }$ with a 95% confidence level. In hand therapy practice, where impaired hands are the major objective, the hand tracking model of the Leap Motion Controller cannot be applied to the finger joint position of injured hands.

**Figure 3:**
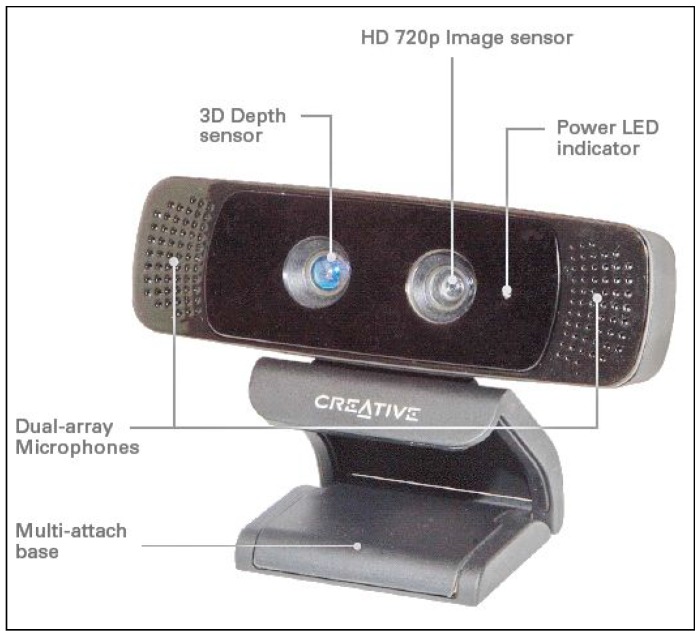
The Creative Senz3D camera.

In this paper, we propose a non-contact measurement scheme for the digits from II–V using a Creative Senz3D camera (Figure 3) to improve the accuracy of current tracking algorithms in estimating finger joint angles. While Kinect is designed for full body capture, the Creative Senz3D is optimized for short-range gesture interaction, and so, it is a better alternative for hand measurements. The remaining parts of this paper are organized as follows. Section 2 presents an overview of the system, while Section 3 presents our approach to improve the accuracy of the system. Section 4 provides computer simulations of the proposed approach. Section 5 and Section 6 present a trial that we have undertaken to validate the proposed system.

## 2. System Overview

**Figure 4:**
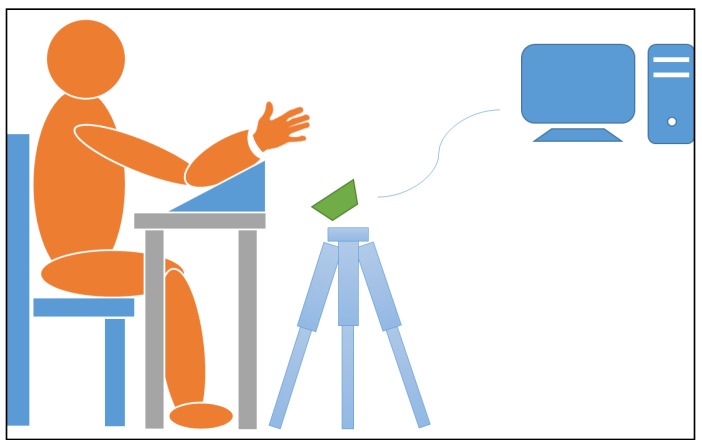
Setup of the measurement system.

The measurement system consists of an affordable optical sensor, Creative Senz3D, and a computer for data acquisition from the optical sensor (Figure 4) and processing. The depth image of the Creative Senz3D has a 640×480 pixel resolution and a refresh frequency of 30 Hz. The base tracking algorithm behind the system was first introduced by Melax [24] and later integrated into the Intel${}^{®}$ Perceptual Computing Software development kit. It uses a convex rigid model to approximate the hand. Each phalangeal bone of the hand is approximated by a convex rigid body, with one rigid body to describe the palm. The rigid hand model is 20 cm long and adjustable to fit most hand dimensions in order to improve accuracy. Tracking information of the hand is optimized with our proposed method represented in Section 3. Our proposed approach improves the accuracy specifically for joint angle measurements, as it is based on the anatomical structure of the hand.

## 3. Accuracy Improvement of Total Active Movement and Proximal Interphalangeal Joint Angles

**Figure 5:**
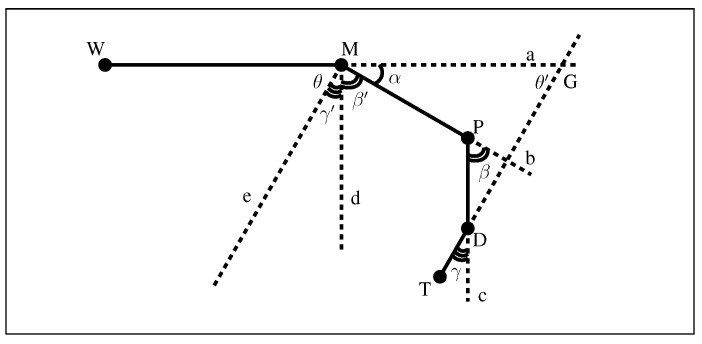
Geometry model of the finger.

To mathematically describe physiological structure (Figure 5), let the wrist joint, metacarpophalangeal joint, proximal interphalangeal joint, distal interphalangeal joint and tip of the finger plane be denoted by the letters *W*, *M*, *P*, *D* and *T*, respectively. Let α,β,γ be the declination angles for the metacarpophalangeal joint, the proximal interphalangeal joint and the distal interphalangeal joint, respectively. Then,

(1) $$\begin{array}{cc} \text{α} & =\arccos\frac{\vec{WM}.\vec{MP}}{\left|\vec{WM}\right|.\left|\vec{MP}\right|} \end{array}$$

(2) $$\begin{array}{cc} \text{β} & =\arccos\frac{\vec{MP}.\vec{PD}}{\left|\vec{MP}\right|.\left|\vec{PD}\right|} \end{array}$$

(3) $$\begin{array}{cc} \text{γ} & =\arccos\frac{\vec{PD}.\vec{DT}}{\left|\vec{PD}\right|.\left|\vec{DT}\right|} \end{array}$$

Furthermore, defined in a global co-ordinate frame, let $e_{i},i\in \left[1,\cdots ,4\right]$ denote the measurement noise of directions of $\vec{WM},\vec{MP},\vec{PD},\vec{DT}$, respectively. Assuming a normal probability distribution, $p\left(e_{i}\right)∼N\left(0,Q_{i}\right)$ for i∈[1,⋯,4], where $Q_{i}$s are indicating the noise power spectral densities.

Using ^ to indicate the measured value and ${}_{0}$ to indicate the actual value, the measured declination angles can be denoted as follows:

(4) $$\begin{array}{ccc} \hat{\text{α}} & = & {\text{α}}_{0}+e_{1}+e_{2}\\ \hat{\text{β}} & = & {\text{β}}_{0}+e_{2}+e_{3}\\ \hat{\text{γ}} & = & {\text{γ}}_{0}+e_{3}+e_{4} \end{array}$$

The concept of total active movement (TAM) has traditionally been used as a means of determining the ROM at the MCP, PIP and DIP joints, and it has been used in the evaluation of hand performance in many studies [28,29,30,31]. The TAM of a finger is defined as:

$$\begin{array}{ccc} \text{S} & = & \text{α}+\text{β}+\text{γ} \end{array}$$

Consequently, the TAM denoted as the interim of declination angles can be stated as,

(5) $$\begin{array}{c} {\hat{\text{S}}}_{1}\;=\;\hat{\text{α}}+\hat{\text{β}}+\hat{\text{γ}}\;=\;e_{1}+2e_{2}+2e_{3}+e_{4}+{\text{α}}_{0}+{\text{β}}_{0}+{\text{γ}}_{0} \end{array}$$

Measuring three phalangeal joint angles to calculate the TAM is invariably subject to the four relevant measurement noise inputs ($e_{1}$, $e_{2}$, $e_{3}$ and $e_{4}$). Noticing that the TAM can be obtained with lesser measurements ($\vec{WM}$ and $\vec{DT}$), and so lesser noise ($e_{1}$ and $e_{4}$), we now look at the geometry model of the finger. Through point *M*, we construct line Md and Me parallel to $\vec{PD}$ and $\vec{DT}$, respectively. Consequently, we have $\hat{\text{β}}$ and ${\hat{\text{β}}}^{'}$ as one pair of corresponding angles and $\hat{\text{γ}}$ and ${\hat{\text{γ}}}^{'}$ as another pair of corresponding angles.

$$\begin{array}{c} \hat{\text{β}}={\hat{\text{β}}}^{'}\\ \hat{\text{γ}}={\hat{\text{γ}}}^{'} \end{array}$$

Let θ be the angle between $\vec{MW}$ and Me. θ can be negative if Me is in the opposite half-plane to the half-plane consisting of *P* and *D*. For the purpose of this paper, we restrict angles α, β and γ to be within the limits of the normal ROM of the human finger joints. Let *G* be the intersection of the lines DT and WM. As $\vec{DT}$ and Me are parallel, $\hat{WGT}$ is equal to θ. Then, it is possible to achieve the TAM through the measurement of $\vec{WM}$ and $\vec{DT}$ as follows.

(6) $$\begin{array}{c} {\text{S}}_{2}\;=\;\text{α}+\text{β}+\text{γ}\;=\;\pi -\text{θ}\;=\;\pi -\hat{WGT} \end{array}$$

Angle $\hat{WGT}$ is dependent only on two measurement noise processes.

$$\begin{array}{c} \hat{WGT}\;=\;\hat{\text{θ}}\;=\;{\text{θ}}_{0}-e_{1}-e_{4} \end{array}$$

The advantage of this approach is that it eliminates noise from the measurement of $\vec{MP}$ and $\vec{PD}$ compared to Equation (5):

(7) $$\begin{array}{c} {\hat{\text{S}}}_{2}\;=\;\pi -\hat{\text{θ}}\;=\;\pi -{\text{θ}}_{0}+e_{1}+e_{4} \end{array}$$

Exploiting this result, we can improve the accuracy of the proximal interphalangeal joint angle. From Equations (5) and (6), we represent the first approach for calculating TAM.

(8) $$\begin{array}{c} {\hat{\text{S}}}_{1}=e_{1}+2e_{2}+2e_{3}+e_{4}+\pi -{\text{θ}}_{0} \end{array}$$

From Equations (7) and (8):

(9) $$\begin{array}{ccc} {\hat{\text{S}}}_{1}-{\hat{\text{S}}}_{2} & = & 2(e_{2}+e_{3}) \end{array}$$

From Equations (4) and (9):

(10) $$\begin{array}{c} {\text{β}}_{0}=\hat{\text{β}}-\frac{{\hat{\text{S}}}_{1}-{\hat{\text{S}}}_{2}}{2} \end{array}$$

Equation (10) shows that we can find the real declination angle for PIP. We choose the estimated value $\tilde{\text{β}}$ of β as described in Equation (10). This is important for the development of a monocular measurement system. There is a high chance of the proximal interphalangeal joint being occluded by the distal inter-phalangeal joint, but our contribution helps to overcome this problem.

In practice, the vectors $\vec{MP},\vec{PD}$ and $\vec{DT}$ are calculated from the point cloud using orthogonal regression for linear fitting. Tracking information of the SDK is only used for segmentation purposes, since the hand tracking algorithm is optimized for speed and recognition rate, rather than the precise bone positions. As the application is focused on the phalangeal joint angle, finding directions of vectors $\vec{MP},\vec{PD}$ and $\vec{DT}$ is crucial. Consider the line **L**, so that the vector $\vec{MP}$ is in the direction vector of **L**:

$$\begin{array}{c} L\left(t\right)=t\vec{D}+A \end{array}$$

where $\vec{D}$ is along the line **L**. Define $X_{i}$ to be the cloud point of the proximal phalangeal bone, then:

$$\begin{array}{c} X_{i}=A+d_{i}\vec{D}+p_{i}{\vec{D}}_{i}^{⊤} \end{array}$$

where $d_{i}=\vec{D}\cdot \left(X_{i}-A\right)$ and ${\vec{D}}_{i}^{⊤}$ is some unit vector perpendicular to $\vec{D}$ with appropriate coefficient $p_{i}$. Define $Y_{i}=X_{i}-A$. The vector ${\vec{N}}^{A}$ from $X_{i}$ to its projection onto the line is:

$$\begin{array}{c} Y_{i}-d_{i}\vec{D}=p_{i}{\vec{D}}_{i}^{⊤} \end{array}$$

The cost function for the least squares minimization is $C\left(A,D\right)=\sum_{i=1}^{m}{∥p_{i}∥}^{2}$. Using Equation (11), the cost function can be rewritten as:

$$\begin{array}{c} C\left(A,D\right)=\sum_{i=1}^{m}\left(Y_{i}^{⊤}\left[I-\vec{D}{\vec{D}}^{⊤}\right]Y_{i}\right) \end{array}$$

Taking the derivative with respect to *A*,

$$\begin{array}{c} \frac{\partial C}{\partial A}=-2\left[I-\vec{D}{\vec{D}}^{⊤}\right]\sum_{i=1}^{m}Y_{i} \end{array}$$

The cost function is minimized when the derivative is zero. A similar approach is used to approximate vectors $\vec{PD},\vec{DT}$.

The direction of the vector $\vec{WM}$ is along the intersection of the finger plane and the palm plane. The palm plane is also approximated using orthogonal regression. The palm plane *p* has a unit normal vector $\vec{N}$ and a central point of the palm **A**. Define $X_{i}$ to be the cloud points of the palm, then:

$$\begin{array}{c} X_{i}=A+\lambda_{i}\vec{N}+p_{i}{\vec{N}}_{i}^{⊥} \end{array}$$

where $\lambda_{i}=\vec{N}\cdot \left(X_{i}-A\right)$ and ${\vec{N}}_{i}^{⊥}$ is some unit vector perpendicular to $\vec{N}$ with appropriate coefficient $p_{i}$. Define $Y_{i}=X_{i}-A$. The vector ${\vec{N}}^{A}$ from $X_{i}$ to its projection onto the palmar plane is $\lambda_{i}\vec{N}$. Then:

$$\begin{array}{c} ∥{\vec{N}}^{A}{∥}^{2}=\lambda_{i}^{2}={\left(N\cdot Y_{i}\right)}^{2} \end{array}$$

The cost function for least squares minimization is $C\left(A,\vec{N}\right)=\sum_{i=1}^{m}\lambda_{i}^{2}$. This can be rewritten as:

$$\begin{array}{c} C\left(A,\vec{N}\right)=N^{⊤}\left(\sum_{i=1}^{m}Y_{i}Y_{i}^{⊤}\right)N=N^{⊤}M\left(A\right)N \end{array}$$

where M(A) is given by:

$$\begin{array}{c} M\left(A\right)=\left[\begin{array}{ccc} \sum_{i=1}^{m}{\left(x_{i}-x_{A}\right)}^{2} & \sum_{i=1}^{m}\left(x_{i}-x_{A}\right)\left(y_{i}-y_{A}\right) & \sum_{i=1}^{m}\left(x_{i}-x_{A}\right)\left(z_{i}-z_{A}\right)\\ \sum_{i=1}^{m}\left(x_{i}-x_{A}\right)\left(y_{i}-y_{A}\right) & \sum_{i=1}^{m}{\left(y_{i}-y_{A}\right)}^{2} & \sum_{i=1}^{m}\left(y_{i}-y_{A}\right)\left(z_{i}-z_{A}\right)\\ \sum_{i=1}^{m}\left(x_{i}-x_{A}\right)\left(z_{i}-z_{A}\right) & \sum_{i=1}^{m}\left(y_{i}-y_{A}\right)\left(z_{i}-z_{A}\right) & \sum_{i=1}^{m}{\left(z_{i}-z_{A}\right)}^{2} \end{array}\right] \end{array}$$

The cost function is in the quadratic form, and the minimum is the smallest eigenvalue of M(A). The corresponding eigenvector $\vec{N}$ that we need to find is normal to the palm plane.

## 4. Simulation

The inference in Section 3 is ascertained through a simulation exercise presented in this section. A configuration of a finger is a set of three declination joint angles of the finger. For each joint, 19 joint angle values of 0–$90^{\circ }$ were evenly generated with a resolution of $5^{\circ }$; hence, there were a total of $19^{3}=6851$ configurations generated for the hand. Random Gaussian noise was added to each joint angle value. The original value of each joint is assumed as the actual value of the joint angle, and the latter value, which was added Gaussian noise, is assumed as the measured value from the tracking algorithm of the camera. These configurations were classed into six groups based on the total active movement value: 0–$45^{\circ }$, 45–$90^{\circ }$, 90–$135^{\circ }$, 135–$180^{\circ }$, 180–$225^{\circ }$ and 225–$270^{\circ }$. The number of configurations for each group is presented in Table 1. After generating data using the above protocol, we had data that cover almost all possible configurations of a human finger. In the next step, we computed the TAM value of each configuration using two methods and compared to the actual value of TAM. In Table 1, the difference between the actual TAM value and the measurements using two methods (${\text{S}}_{1}$ and ${\text{S}}_{2}$) mentioned above is denoted by,

$$\begin{array}{ccc} \Delta {\text{S}}_{1} & = & |{\hat{\text{S}}}_{1}-{\text{S}}_{0}|\\ \Delta {\text{S}}_{2} & = & |{\hat{\text{S}}}_{2}-{\text{S}}_{0}| \end{array}$$

**Table 1:** Number of configurations for 6 groups of total active movement (TAM).

Group	Quantity	$\Delta {\text{S}}_{1}$	$\Delta {\text{S}}_{2}$	$\Delta {\text{β}}_{1}$	$\Delta {\text{β}}_{2}$
0–$45^{\circ }$	165	13.7	5.9	6.1	0
45–$90^{\circ }$	975	12.3	5.7	5.5	0
90–$135^{\circ }$	2154	12.5	5.6	5.6	0
135–$180^{\circ }$	2235	12.1	5.5	5.6	0
180–$225^{\circ }$	1110	13	5.8	5.8	0
225–$270^{\circ }$	220	12.7	5.4	5.6	0

Overall, the values of $\Delta {\text{S}}_{1}$ are larger than the values of $\Delta {\text{S}}_{2}$ in the same group. The mean values of 6851 configurations for $\Delta {\text{S}}_{1}$ and $\Delta {\text{S}}_{2}$ are $12.47^{\circ }$ and $5.62^{\circ }$, respectively. From these figures, implementing the proposed approach to find the TAM value can generally reduce the error of measurement of TAM from $12.47^{\circ }$ down to $5.62^{\circ }$, which is significant. Similarly, the differences between the actual PIP joint angle ${\text{β}}_{0}$ and the initial measurement $\hat{\text{β}}$ is denoted as $\Delta {\text{β}}_{1}$; the difference between the actual PIP joint angle ${\text{β}}_{0}$ and the estimated value $\tilde{\text{β}}$ is denoted by $\Delta {\text{β}}_{2}$, *i.e.*,

$$\begin{array}{c} \Delta {\text{β}}_{1}=\hat{\text{β}}-\beta_{0}\\ \Delta {\text{β}}_{2}=\tilde{\text{β}}-{\text{β}}_{0} \end{array}$$

**Figure 6:**
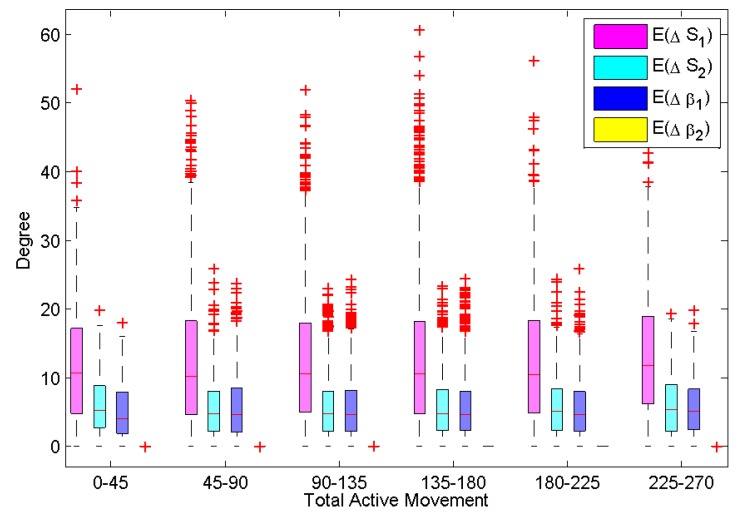
Simulation results for the accuracy improvement of the TAM and proximal interphalangeal joint (PIP).

The mean values of 6851 configurations for $\Delta {\text{β}}_{1}$ is $5.64^{\circ }$, while the one for $\Delta {\text{β}}_{2}$ is $0^{\circ }$. This result implies that the mean error of initial measurement is $5.64^{\circ }$, and the value of approximately $0^{\circ }$ is the difference between the actual ${\text{β}}_{0}$ and the estimated $\tilde{\text{β}}$ obtained by Equation (10), thus confirming our assertions. Figure 6 represents further details of the statistical characteristics, including the median, quartiles and whiskers. In summary, this simulation shows the reductions in error if the proposed approach is employed on top of the camera measurements.

## 5. Trial Procedure

In this section, we present a validation procedure to ascertain the reliability of the system. The final target of this procedure was to examine if the system can be used in clinics to replace the traditional universal goniometer. The experimental procedure was approved by Deakin University Human Ethics Advisory Group (HEAG), and all participants provided their written informed consent to participate. Two professional hand therapists conducted this trial using the universal goniometer. Although human ratings can be subjective and may not possess a degree of accuracy, it is the current practice. The procedure was conducted following the clinical recommendations [32]. The dorsal measurement technique, which was proven to be as reliable as lateral placement [33], was used during the procedure.

**Figure 7:**
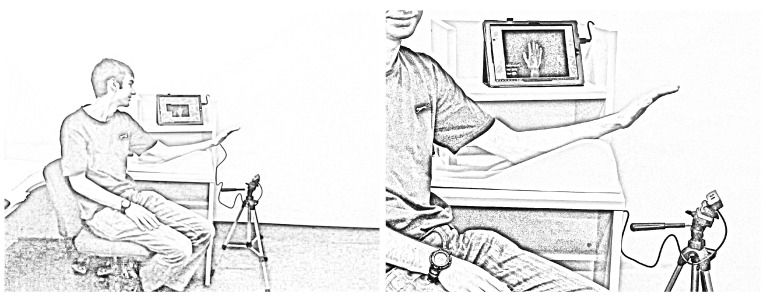
The left hand of the man was measured by our system.

Forty participants were recruited to participate in the trial. There were 35 females and 5 males in the study, with the mean age being 29.3 years (σ=11.5). Thirty five subjects were right hand dominant, and 5 subjects were left hand dominant. None of the participants reported having previous hand injuries that would have impacted significantly on their current ROM. Participants were seated at a table with their arm on the hand elevator and elbow supported on the table. This position put the shoulder in an approximately 45–80${}^{\circ }$ flexion, the elbow in approximately 40–60${}^{\circ }$ flexion and the wrist in a neutral, pronated position. The Creative Senz3D camera was mounted on a tripod and placed below, in front of the participant’s hand, as depicted in Figure 7. A training trial was conducted prior to the commencement of the research to practice the protocols and logistics of the study.

**Figure 8:**
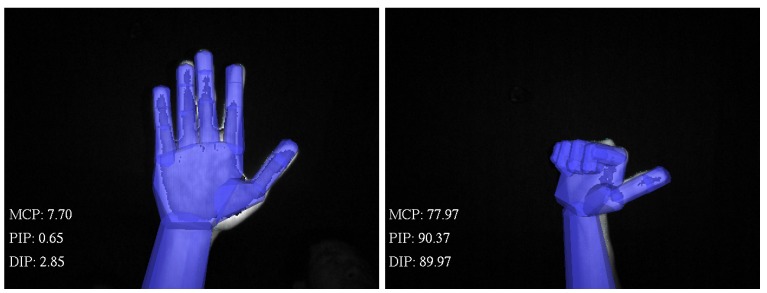
Extension and flexion positions of the hand in the tracking application.

The Creative Senz3D camera was connected to a computer with Intel Core i7-3740QM 2.7Ghz CPU, 8GB RAM, Nvidia NVS 5400M VGA with Windows 7 64-bit installed. The Intel${}^{®}$ Perceptual Computing SDK was installed for aiding the hand tracking stage. The declination joint angles of the hand were computed from the hand model when the model precisely matched the subject’s hand (Figure 8). Then, these values were employed to compute the estimation of the TAM and PIP angle values utilising our proposed approaches.

The universal goniometer is a standard six-inch clear plastic instrument that can measure 0–180${}^{\circ }$ angles to a precision of 5${}^{\circ }$. It is one of the preferred goniometers for hand joint measurements [34]. The long edge of the goniometer was used to measure the MCP and PIP joints, and the short edge was used to measure the DIP joint. The two clinicians used the same goniometer.

The participants were recruited using a non-probability convenience sampling method. Participants provided their written informed consent to participate prior to data collection. Participant names were encoded to ensure all information was anonymous.

Two standardised positions of the hand, extension and flexion, were measured by using two different tools, and the results were compared. The participant’s hands were measured firstly by the proposed system and secondly by the goniometer. Placing the hands in front of the camera, the participants were requested to “straighten your fingers while keeping them slightly separated”. When the hand model matched the participant’s hand, *i.e*., the error between the hand model and the depth image was less than a threshold, information was captured twice by pressing a button on the keyboard. The time required between the two pressings was one second. The hand then remained in the same position for the first clinician to measure MCP, PIP and DIP joints using the universal goniometer followed by the second clinician. For each group of ten participants, pre-allocated fingers from Digits II–V were measured. The time between the two measurements by the clinicians was approximately one second, including the time allowed for handing the goniometer. Each participant had two separate forms for the two clinicians to record the results. A stop watch was used to record the measurement time, and these numbers were recorded on the results form. In case the prescribed procedure was not followed, the data were deleted, and the process was repeated. Following the measurement of the hand in the extended position, the participants were able to relax their hand. Subsequent to straightening the fingers again so that the capture system is able to recognise the hand, participants were asked to “bring your fingers into a fist position while keeping your thumb fully extended”. Similar to the measurement for the case of extension, the hand was measured by the proposed system and followed by the universal goniometer. The procedure was repeated for the left hand. Participants were thanked for participating in the trial and were informed that the overall results would be made available in the future.

## 6. Results

### 6.1. Concurrence Validity

**Figure 9:**
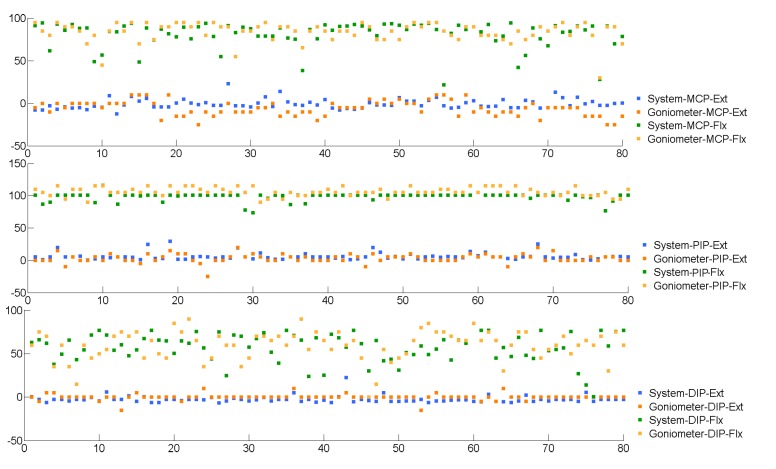
The ROM of MCP, PIP and DIP measured by the proposed system and a therapist. The values from 1–40 and from 41–80 on the x-axis are the left hands of 40 subjects and the right hands of 40 subjects, respectively.

We established the validity of the proposed approach by comparing to the scores obtained from a well-established measurement procedure for the same cohort of subjects; *i.e*., there exists a consistent correlation between the scores from the two contrasting and independent measurement procedures [35]. Concurrent validity is often determined using the Pearson product-moment correlation. Following the trial, a total of 480 angles were measured by each tool. The procedure was limited to one finger per hand per subject, excluding the thumb. For each angle, which is at the joint in a finger, the variable $X=x_{i}$ is the measurement of the proposed system, and the variable $Y=y_{i}$ is the measurement of the universal goniometer. The correlation between the two variables *X* and *Y* was r=0.95. At the joint level, the correlation between the proposed system and the universal goniometer is $r_{MCP}=0.96$, $r_{PIP}=0.98$ and $r_{DIP}=0.87$. The relationship between the proposed approach and the manual goniometer-based measurements indicates that all of the variables demonstrate a higher degree of correlation (r>0.85). The root mean square errors (RMSE) of the difference between the proposed system and universal goniometer are calculated and depicted in Table 2. The absolute values of the ROM of three joints measured by the proposed system and a therapist were plotted in Figure 9.

**Table 2:** Comparison between the proposed system and universal goniometer. MCP, metacarpophalangeal joint; DIP, distal interphalangeal joint; PIP, proximal interphalangeal joint.

Variable	RMSE (${}^{\circ }$)
MCP	14.8
PIP	12.6
DIP	11.4

### 6.2. Internal Reliability

The results of two measurements taken by the same tool, either the proposed system or the universal goniometer, were compared to infer the intraclass correlation coefficient (ICC) with a 95% confidence interval. According to Portney *et al.* [36], the ICC value of above 0.75 is considered as good reliability, while a good clinical measurement should have a value of above 0.9. The overall ICC of the proposed system was 0.998% at a 95% confidence level. In comparison, the overall ICC of the universal goniometer is 0.994% at a 95% confidence level. The results of the analysis on extension and flexion and at the joint level are shown in Table 3. The results show that the flexion has better ICC values than extension in both the proposed system and the universal goniometer. At the joint level, the DIP demonstrated the lowest ICC values in both the proposed system and the universal goniometer, but these values are still considered to have clinically good reliability (ICC >0.9).

**Table 3:** The internal reliability of the proposed system. ICC, intraclass correlation coefficient.

Variable	Proposed System		Universal Goniometer	
	ICC	Confidence Interval (95%)	ICC	Confidence Interval (95%)
MCP	0.999	0.999–1	0.995	0.993–0.996
PIP	0.999	0.999–1	0.998	0.997–0.998
DIP	0.993	0.990–0.996	0.986	0.980–0.990

**Figure 10:**
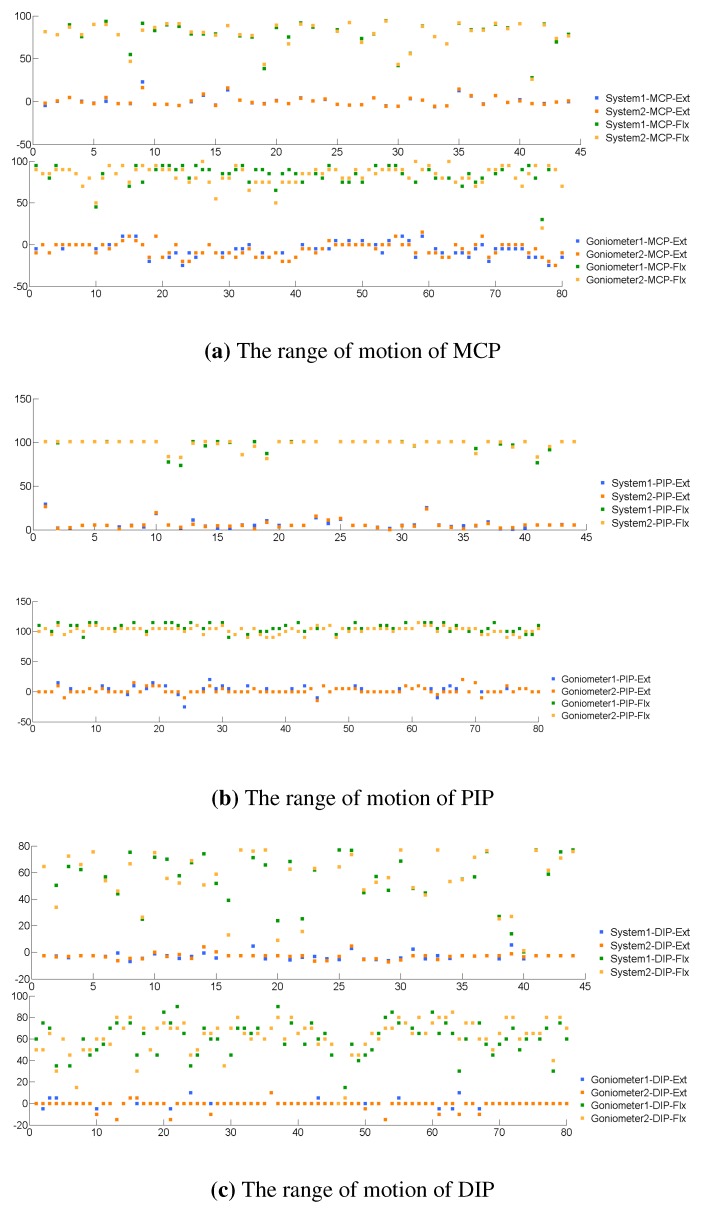
The range of motion (ROM) of MCP, PIP and DIP measured twice by either the proposed system or therapists. The values from 1–40 and from 41–80 on the x-axis are the left hands of 40 subjects and the right hands of 40 subjects, respectively.

To determine the difference of repeated measurements, the results of two measurements taken by the same tool, either the proposed system or the universal goniometer, were compared using the RMSE analysis metric. The data between two repeated measurements by either the proposed system or therapists are plotted in Figure 10. Due to the limitation in experimental design, the results of the proposed system were calculated on 22 subjects (n=264), and the results of the goniometer were calculated on 40 subjects (n=480). A *t*-test indicated that the internal difference between the proposed system and the universal goniometer was statistically significant. Further details of the analysis at the joint level are shown in Table 4. In three joints, the DIP often had a greater difference and greater variance between repeated measurements than the two remaining joints. The result analysis of repeated measurements clinically and statistically demonstrates the reliability of the proposed system.

**Table 4:** Comparison of the consistency of the proposed system and the universal goniometer.

Variable	Proposed System RMSE (${}^{\circ }$)	Universal Goniometer RMSE (${}^{\circ }$)
MCP	2.2	6.8
PIP	2.3	5.3
DIP	6.4	7.9

### 6.3. Time Efficiency

For each position of the hand, the proposed system was able to capture the joint angles of 12 joints of the hand, whereas for a manual assessor, only three joints were measured. Therefore, the proposed system needs only one measurement for each extension or flexion position, while the manual assessor needs five measurements for each extension or flexion position. The time of measurement was divided by the number of joints measured. Consequently, the measurement time using the proposed system was divided by 12, and the measurement time using a manual assessor was divided by three. The mean time per joint of the proposed system was calculated on 16 subjects, and the mean time per joint of the universal goniometer was calculated on 40 subjects. The mean time per joint of the proposed system was 2 s (σ=0.9). The mean time per joint of the universal goniometer was 4.4 s (σ=2). A *t*-test indicated that the difference in measurement time between the proposed system and the universal goniometer was statistically significant. The details of the measurement times are reported in Table 5. As depicted in Table 5, the proposed system completed the measurements 2–4 s quicker than the therapists with a universal goniometer.

**Table 5:** Average time of measurement per joint.

Item		Mean Time per Joint (s)	SD (s)
Proposed system		1.2	0.9
Universal goniometer		4.4	2.0

## 7. Discussion

The correlation between the proposed system and the universal goniometer is strong enough to confirm that the proposed system provides similar measurements of finger ROM to the universal goniometer. The mean difference between the proposed system and the universal goniometer of 9.53${}^{\circ }$ is close to previous studies in which the mean difference between two therapists using a universal goniometer is 7–9${}^{\circ }$. From these analyses, it can be inferred that the proposed system is a valid tool to use in finger ROM measurement for Digits II–V, although the clinical differences are greater than the 5${}^{\circ }$ required for clinical significance.

The ICC of the proposed system was found to be very strong (ICC =0.998, ICC${}_{ext}=0.974$, ICC${}_{flx}=0.99$). The universal goniometer measurement also showed very strong ICC coefficients, although less than the proposed system. This implies that the proposed system is capable of reproducing almost the same results for the same joint angle. The consistency of the system enables it to be more accepted as a standard measurement tool. The analysis also demonstrates that the mean difference of the proposed system in two repeated measurements is 1.94${}^{\circ }$. This number is less than 5${}^{\circ }$ of clinical significance, thus inferring that the proposed system is more reliable when being used in a test-retest scenario, which is common in clinical practice.

Experimental results demonstrated that our system is much more effective in terms of operating time than the manual process, saving up to four seconds per joint. In practice, when measuring all joints of the hand, the system is capable of saving up to several minutes due to the additional time required for data entry.

The findings of this experiment may not be generalized to a clinical setting for a number of reasons. As the target of this study was to introduce a non-contact ROM measurement for the hand, we only recruited healthy subjects, without oedema or wounds, to simplify the procedure. In addition, two therapists had to memorize the results of three joint measurements before entering the numbers to the report, thus increasing the chance of observer error. Indeed, the camera is new to the market and slightly more expensive than the widely-used manual goniometers. Wider use of the proposed technology will also help to reduce the cost of these new devices. Lastly, despite endeavouring to ensure that the subject’s hand remained in the same position, it was impossible to maintain the position of the hand for 30 s without any change, and the force of making a fist can inadvertently contribute to measurement error. Because of short length of phalangeal bone, even a small movement of the hand can largely impact the reading of both the therapists and the proposed system.

The improvement of ROM is considered a key aspect in hand therapy practice. Busy clinics often have their clients treated by different therapists, which raises the issue of accuracy and consistency in measuring ROM among therapists. An automated, non-contact system replacing humans in measurement tasks ensures the consistency of measurements and the effectiveness of treatments. In order to be used as an effective ROM tool, the measurements need to be reliable and valid [37]. The accuracy of current devices, such as manual goniometers or electro-goniometers, experiences a higher degree of subjectivity based on the assessor. In certain situations, it can be uncomfortable for the patient and increase the risk of infections upon contact. The development of the proposed system was motivated by the demand for a standardized, reliable, objective and time-efficient system for measuring phalangeal ROM. As motion capture devices are widely considered and investigated, sensors in the future with a higher resolution, a higher sampling rate and less noise can further improve the performance of the system. The research can be considered as a proof of concept for the potential use of the system in clinical settings. It is expected to see variants of the proposed system in clinical settings in the next few years.

The use of advanced technology reduces the cost of healthcare and shortens appointment sessions [38] without compromising the quality of care. In recent decades, computer-based evaluation systems for hand therapy have evolved, and their use in hand therapy clinics is becoming a common practice. Computer-based evaluation systems like ours are in demand and are destined to be integrated into existing healthcare systems and hospitals to enhance rehabilitation processes. The portability and convenience of our system allows it to be used in different locations, enabling it to have a significant impact on tele-rehabilitation.

## 8. Conclusions

Use of technology in healthcare reduces cost and enhances quality of service and patient comfort. Our proposed system provides clinicians with an innovative and effective solution to evaluate ROM of the hand. More importantly, the non-contact and faster measurements help improve the patient comfort, as the time of measurement is shortened significantly. In addition, the proposed approach can be used in many other potential applications involving quantitative assessment of finger functionality. A non-contact phalangeal measurement system has the potential to become a standard facility in assessing hand function, offering an additional choice in clinical use. An expansion of this system to tele-health and other e-health applications is a necessary step to integrate the system into hospital and to enhance client satisfaction.
